# Influence of Sex and Muscarinic Activity on Memory Retrieval in Mouse Model of Traumatic Brain Injury

**DOI:** 10.3390/brainsci13010108

**Published:** 2023-01-06

**Authors:** Habiba Rashid, Touqeer Ahmed

**Affiliations:** 1 Neurobiology Laboratory, Department of Healthcare Biotechnology, Atta-ur-Rahman School of Applied Biosciences, National University of Sciences and Technology, Sector H-12, Islamabad 44000, Pakistan; 2 Department of Anatomy, Institute of Basic Medical Sciences, Khyber Medical University, Hayatabad Phase-V, Peshawar 25100, Pakistan

**Keywords:** sex dimorphism, donepezil, scopolamine, spatial memory, contextual fear, weight drop TBI

## Abstract

Traumatic brain injury (TBI) is a serious global risk factor leading to the onset of cognitive impairment and neurodegenerative diseases. Cognitive and memory impairment following a TBI is associated with the dysregulation of cholinergic neurotransmission in the brains of subjects. The extent of memory impairment following a TBI is linked with the sex of the subject. This study aimed to identify the sex-dimorphic role of muscarinic cholinergic modulation in neurological functioning and episodic memory retrieval in a mouse model of TBI. Balb/c mice were divided into four groups of males and four groups of females (i.e., Sham, TBI, TBI + Scopolamine 1 mg/kg, and TBI + Donepezil 1 mg/kg). After training with the Morris water maze test and fear conditioning, all groups were subjected to brain injury (7.84 × 10^−5^ J impact force) except for the Sham mice. Following brain injury, scopolamine or donepezil was administered to the respective groups for 5 days. Acute scopolamine immediately after brain trauma showed a neuroprotective effect in the males only, while subchronic donepezil significantly impaired neurological functioning in both sexes. Subchronic scopolamine and donepezil treatment reversed the TBI-induced retrograde amnesia for spatial memory in male mice. Contextual fear memory retrieval was not affected by the TBI and treatments in both sexes. Thus, we concluded that the sex-dimorphic response of the muscarinic receptors in TBI-induced memory impairment depends on the type of memory. This study highlights the potential for therapeutic modalities in TBI subjects.

## 1. Introduction

Traumatic brain injury (TBI) is a brain insult in response to a spontaneous force that causes changes in normal brain functioning. It is a leading cause of mortality and neurological disability [1]. TBI is associated with significant cognitive deficits affecting memory, information processing, and behavior [1]. There are multiple causes of TBI; the most common include traffic accidents, falls, and blast injuries [2,3]. The incidence of TBI is three times greater in men than in women [3,4]. The biological mechanism of injuries, particularly TBI, involves inflammation [5], edema [6], dysregulation of neurotransmitter systems, oxidative stress [7], and mitochondrial dysfunction [8]. TBI is associated with an increased risk of developing neurodegenerative diseases, with an established role in the early onset of Alzheimer’s disease [9]. Sex is an important factor associated with post-TBI cognitive outcomes [10,11]. Experiments have revealed the neuroprotective potential and better recovery from mild TBI-induced cognitive effects in female rodents under physiological conditions but not in males [12,13]. The exact mechanism of this neuroprotection in females compared to males is not completely understood. The endogenous hormones in females are suggested to pose neurological protection following a TBI in young female rats but not in males [13].

TBI affects learning and memory processes by impairing the acquisition, consolidation, and retrieval of memory [14]. TBI induces a memory deficit by impairing cholinergic transmission, which is the key player in learning and memory processes [15,16,17,18]. A deduction in acetylcholine synthesis and release in the hippocampus is reported in animals following mild TBI [19,20]. Mild TBI has been linked with the downregulation of hippocampal muscarinic cholinergic receptors following the insult [21,22]. When considering the cognitive effects of TBI via cholinergic system impairment, acetylcholinesterase inhibitors are used to treat cognitive deficits in patients with TBI [23,24]. Donepezil, an acetylcholinesterase inhibitor, has emerged as a potential therapeutic candidate for treating post-traumatic cognitive disorders [25,26,27,28]. Despite these findings revealing the therapeutic potential of muscarinic receptor modulation in post-TBI cognitive outcomes, the existing data are greatly generated from studies on male subjects, and sex differences are understudied.

By keeping the importance of sex dimorphism in the post-TBI cognitive outcomes and the potential role of muscarinic receptor modulation in treating TBI-induced memory impairment in view, this study was designed to determine the influence of sex on the muscarinic receptor’s role in reversing TBI-induced neurological and memory recall deficits in young mice. Morris water maze and context retention testing were used to assess memory retrieval function. Neurological severity scoring was performed to determine neurological, locomotor, and exploratory functioning. Sub-chronic muscarinic modulation displayed a sex-dimorphic effect in reversing mild TBI-induced memory retrieval impairment in a task-dependent manner.

## 2. Materials and Methods

### 2.1. Animals

Male and female Balb/c mice (3–4-month-old) were used in the study. All the experimental protocols were approved by the Internal Review Board of Atta ur Rahman School of Applied Biosciences (ASAB), National University of Sciences and Technology (NUST), with the specific reference number (IRB No: IRB-62). All the experiments were carried out in accordance with the National Institutes of Health guide for the care and use of laboratory animals (NIH Publications No. 8023, revised 1978). All efforts were made to minimize the suffering caused to the animals. Mice were purchased from NIH Islamabad, Pakistan, and kept in the Laboratory Animal House of ASAB-NUST, Islamabad. Male and female mice were housed in separate cages under natural light and dark cycles, with free access to food and water. Mice were housed in groups of six to eight animals per cage.

### 2.2. Experiment Design

**Figure 1:**
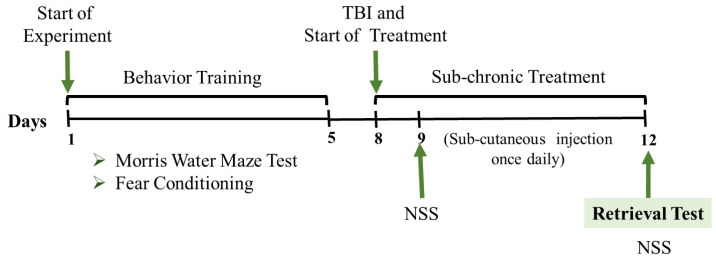
Scheme of the experimental design. TBI was induced after 5 days of behavior training, and memory retrieval was performed on day 12. TBI: traumatic brain injury, NSS: neurological severity scoring.

We explored the sex-dimorphic effect of TBI on relatively remote memory retrieval and the response of sub-chronic scopolamine and donepezil in a mouse model of TBI. Balb/c mice were randomly divided into four male and four female groups. The groups were Sham (no trauma), TBI (trauma), TBI + Scopolamine (trauma with subcutaneous 1 mg/kg scopolamine), and TBI + Donepezil (trauma with subcutaneous 1 mg/kg donepezil). All the groups had *n* = 7 mice per group except the male TBI + Scopolamine group, which had *n* = 8. The experiment scheme comprised 12 days (Figure 1). Days 1–5 were the training days in which the mice were trained for spatial memory formation using the Morris water maze testing. Fear conditioning was performed on day 5. No behavior or treatment was carried out on days 6 and 7. On day 8, all the groups were anesthetized (100 mg/kg ketamine and 7 mg/kg xylazine), and TBI was induced, except for in the Sham mice. From day 8 to day 12, scopolamine or donepezil was administered to the respective groups. Neurological severity scoring was recorded on day 9 (24 h post-trauma and first drug administration) and day 12 (96 h post-trauma). On day 12, the test day, 30 min after drug administration, memory retrieval tests were performed. After the retrieval tests, the brains were collected by decapitating mice under anesthesia (Figure 1). In order to prevent the influence of one task on the memory of another, a minimum of 30 min gap was given between the two consecutive memory tasks. On day 5, the sequence of experiments was as, first we performed the last training trial of the Morris water maze test and then 40 min later, fear conditioning was performed. On the test day, the sequence of the experiment was NSS, followed by probe trials, followed by context retention. A minimum of 30 min and a maximum of 60 min interval was given to each group between the two consecutive memory and behavior tasks.

### 2.3. Drugs

The following drugs were used: scopolamine hydrobromide (sc- 296372, Santa Cruz biotechnology, TX, USA) and donepezil HCl (Genome Pharmaceutical (Pvt) Ltd. Punjab, Pakistan). Drugs were prepared in 0.9% saline and kept at 4 °C. Subcutaneous route was used for drug administration.

### 2.4. Behavior Tests

#### 2.4.1. Morris Water Maze Test

Morris water maze testing was performed as described previously by our lab [29]. All the mice were trained to find and sit on a hidden platform in the water maze tank. A total of 25 training trials were conducted from day 1 to day 5. Swim latency to reach and sit on the platform was recorded using a stopwatch. A probe trial was carried out on the test day, i.e., day 12, in which the platform was removed from water maze tank, and the mice were introduced to the tank for 90 s. The probe trial of each animal was recorded using a video camera for later analysis. Videos were manually analyzed for three parameters: time spent by mice in the target quadrant in seconds, number of times mice entered the target quadrant, and the number of crossings over the platform location. A partial blind analysis of the Morris water maze training and probe trials was performed, one experimenter was aware of the groups and treatment, while the second experimenter was unaware of the treatment given to mice.

#### 2.4.2. Fear Conditioning and Context Retention

On day 5, fear conditioning was carried out as previously described by our lab [30]. Briefly, mice were subjected to five 30 s auditory tones (70 dB, 3000 Hz), co-terminated with a 1 s, 0.3 mA foot shock in the conditioning chamber. The inter tone interval was two minutes, and the mice were moved to their home cage after fear conditioning. Context retention was performed on day 12 by placing the mice in the same conditioning arena for 330 s without giving an auditory tone or foot shock. Freezing during the fear conditioning and retrieval sessions were recorded using Any Maze software. Fear retrieval was calculated in terms of the percent freezing response using the following formula:

*Percent freezing = total time of freezing in seconds*/330 s

### 2.5. Traumatic Brain Injury

To determine the effects of TBI and muscarinic signaling on memory retrieval, on day 8, injury was induced by using the weight drop method [31]. Weight drop apparatus consisted of a steel tube fixed vertically in a stand. A blunt-ended iron rod (200 mg in weight and 2 mm in diameter) was dropped through the steel tube from 4 cm height to hit the skull with 7.84 × 10^−5^ J impact force, inducing experimental TBI. The force was measured as:

*Force = (m × g) × d*

where “*m*” is mass, “*g*” is acceleration due to gravity (9.8 m/s^2^), and “*d*” is height/distance.

Mice were anesthetized using ketamine (100 mg/kg) and xylazine (10 mg/kg), and the fur was shaved from head using scissors. A longitudinal incision was made on the scalp skin to expose the skull. Mice were then placed on the platform of the weight drop apparatus. The incision was manually exposed, and the rod was dropped from 4 cm height to hit in between the bregma and lambda. Three hits were given to each animal to induce TBI. After the injury, the animals were removed from the apparatus, and an incision was closed using silk suture. Mice were placed back in their home cages and were allowed to recover. The Sham mice passed through the same procedure of anesthesia and scalp incision but without receiving the induced trauma.

### 2.6. Neurological Severity Score

The extent of neuronal damage was accessed after 24 h of injury (day 9) and on the retrieval day (day 12) prior to the retrieval tests by evaluating the neurological severity score (NSS). The NSS score is directly related to the severity of neuronal damage. The scale ranges from 0–30, where 0 represents normal neurological functioning and 30 shows maximum neurological deficit. The successful completion of a task scores 0, while the maximum score represents the failure to complete a task (Table 1). An NSS score between 0–10 represents mild TBI [32], which was the case in our study.

### 2.7. Evaluation of Cerebral Edema

Edema was evaluated by measuring the water content in the injured brain, as described previously [31], with slight modifications. After completion of all tasks on day 12, the mice were anesthetized, decapitated, and the whole brains were harvested. The brains were placed in respectively labeled Eppendorf tubes and weighted to yield their wet weight. The Eppendorf tubes were placed in an oven at 70 °C for 24 h and reweighted to yield the dry weight. Cerebral edema was calculated in terms of percent water content using the following formula:

Percent water content=((Wet weight−Dry weight)/Dry weight)×100

### 2.8. Statistical Analysis

Statistical analysis and graphical plotting were performed using GraphPad Prism (version 5.0). Results were analyzed using one-way ANOVA, followed by posthoc Bonferroni’s test. Data are represented as mean ± SEM; *p* < 0.05 was considered significant.

## 3. Results

### 3.1. Neurological Severity Scoring (NSS)

Neurological severity scoring was used to assess the extent of neurological damage by evaluating the motor functions and reflexes of the mice that underwent a TBI. We recorded the NSS 24 h and 96 h post-TBI.

**Figure 2:**
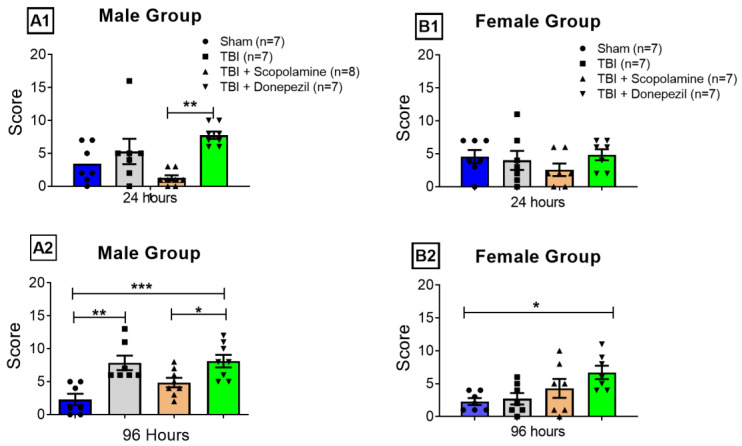
Neurological severity scoring of TBI mice. (**A1**,**B1**) show the neurological scores of the male and female mice 24 h post-trauma, respectively. (**A2**,**B2**) show the neurological severity scores of the male and female mice, respectively, 96 h after trauma. Data were analyzed using one-way ANOVA and are presented as mean ± SEM. * *p* < 0.05, ** *p* < 0.001 and *** *p* < 0.0001. TBI: traumatic brain injury, *n*: number of mice per group.

A significant effect post-TBI muscarinic modulation was found 24 h after trauma induction in the males (One-way ANOVA: F (3, 26) = 6.76; *p* = 0.001; Figure 2(A1)) but not in the female mice (One-way ANOVA: F (3, 24) = 0.88; *p* = 0.46; Figure 2(B1)). There was no difference in the scores of the male Sham (3.42 ± 1.08) and TBI (5.28 ± 1.92) groups 24 h after surgery. Scopolamine administration immediately following the trauma in the males showed a neuroprotective effect, showing the lowest score (TBI + Scopolamine = 1.25 ± 0.41) when compared to all groups 24 h after injury. While donepezil administration in the males immediately after trauma induced a neurological deficit, evident by a higher score (7.75 ± 0.55) compared to the TBI + Scopolamine group (Bonferroni posthoc: *p* < 0.05), Sham and TBI groups (Figure 2(A1)). On the other hand, the female mice (24 h after trauma) showed a similar score irrespective of treatment (Sham = 4.571 ± 0.99; TBI = 4 ± 1.44; TBI + Scopolamine = 2.57 ± 0.94; TBI + Donepezil = 4.85 ± 0.82; Figure 2(B1)).

On the test day, NSS was performed after subchronic muscarinic modulation (96 h post-trauma). The one-way ANOVA analyses revealed significant effects post-TBI muscarinic modulation for NSS in the male (F (3, 26) = 8.88; *p* = 0.0003; Figure 2(A2)) and female mice (F (3, 24) = 3.88; *p* = 0.02; Figure 2(B2)). Neurological severity was scored significantly higher in the TBI (7.85 ± 1.10, *p* < 0.005) and TBI + Donepezil (8.12 ± 0.95, *p* < 0.0001) male groups when compared to the Sham group (2.28 ± 0.86). Unlike the 24 h post-trauma scores, the NSS score of the male TBI + Scopolamine (4.87 ± 0.71) 96 h post-trauma group was greater than the Sham group but was significantly less than the TBI + Donepezil (*p* < 0.05, Figure 2(A2)) group. Unlike the males, there was no significant difference between the Sham (2.28 ± 0.52), TBI (2.71 ± 0.86), and TBI + Scopolamine (4.28 ± 1.44) neurological severity recorded 96 h post-trauma in the females. However, female TBI + Donepezil (6.71 ± 1.01) showed a significantly higher neurological severity score compared to the Sham group (*p* < 0.05, Figure 2(B2)).

In order to identify a clear injury effect, we pooled the NSS scores for both sexes; we did not find any significant differences between the Sham vs. TBI groups at 24 h post-trauma (Sham 24 = 4.00; TBI 24 = 4.63), while the 96 h postinjury score of the TBI animals was significantly greater than the Sham group (Sham 96 = 2.28; TBI 96 = 5.28; *p* = 0.01).

### 3.2. Effect of TBI and Muscarinic Receptors on Contextual Fear Memory Retrieval

**Figure 3:**
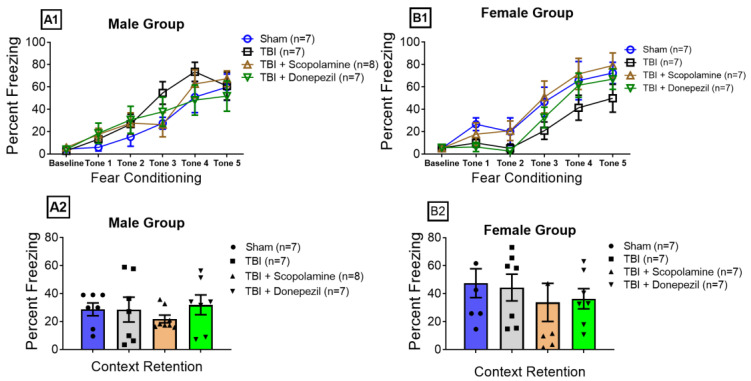
Effect of muscarinic modulation and TBI on fear memory retrieval. Graphs (**A1**,**B1**) represent the fear conditioning in the male and female mice, respectively. (**A2**,**B2**) show the retrieval of fear memory in the male and female mice, respectively, on day 12. Data are presented as mean ± SEM. TBI: traumatic brain injury; *n*: number of mice per group.

The male and female mice were trained for fear conditioning on day 5. All the groups acquired sufficient fear memory, evidenced by an increased percentage in the freezing response of the male (Figure 3(A1)) and female groups (Figure 3(B1)) on the last tone, i.e., tone 5. Baseline freezing was recorded 30 s before the onset of the first tone.

One-way ANOVA analyses revealed that TBI alone or in combination with the cholinergic drugs did not affect contextual fear retrieval in the male (F (3, 25) = 0.51, *p* = 0.678, Figure 3(A2)) and female mice (F (3, 24) = 0.387, *p* = 0.762, Figure 3(B2)). All the male (Sham: 28.68 ± 4.60%, TBI: 28.52 ± 8.89%, TBI + Scopolamine: 21.79 ± 2.78%, and TBI + Donepezil: 31.92 ± 7.10%) and female groups (Sham: 47.47 ± 10.32%, TBI: 44.39 ± 9.54%, TBI + Scopolamine: 33.76 ± 13.58%, and TBI + Donepezil: 36.36 ± 7.18%) showed similar freezing during context retention.

### 3.3. Effect of TBI and Muscarinic Receptors on Spatial Memory Retrieval

**Figure 4:**
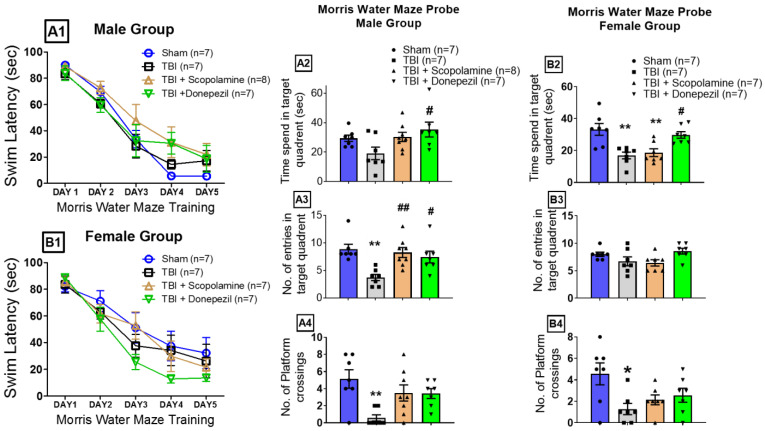
Effect of post-TBI muscarinic modulation on spatial memory retrieval. Graph (**A1**) and (**B1**) represent the Morris water maze training data for the male and female groups, respectively. (**A2**–**A4**) represent the time spent in the target quadrant, entries into the target quadrant, and platform location crossings, respectively, by the male mice during probing. (**B2**–**B4**) show the times spent in the target quadrant, the entries into the target quadrant, and the platform location crossings, respectively, by the female mice during spatial memory retrieval in the probe trial. Data are presented as mean ± SEM. * *p* < 0.05 and ** *p* < 0.001 compared to Sham; ^#^ *p* < 0.05 and ^##^ *p* < 0.001 compared to TBI.

The male and female mice efficiently learned the location of the hidden platform during the Morris water maze training, as evidenced by a reduction in swim latency for the male and female groups on day 5 (Figure 4(A1,B1)).

On the test day, there was a significant difference in the time spent in the target quadrant by the male (one-way ANOVA: F (3, 25) = 3.13, *p* = 0.04; Figure 4(A2)) and female groups (one-way ANOVA: F (3, 24) = 9.21, *p* = 0.0003; Figure 4(B2)). TBI in the males reduced the time spent in the target quadrant in the TBI group (19.14 ± 4.27 s) when compared to the Sham group (29.33 ± 2.16 s). Subchronic scopolamine administration slightly (30.31 ± 3.12 s, Bonferroni posthoc: ^#^ *p* < 0.05), while donepezil significantly reversed the TBI-induced reduction in time spent in the target quadrant (Figure 4(A2)). TBI significantly reduced the time in the target quadrant regarding the female mice (16.93 ± 2.07 s) when compared to Sham mice (33.27 ± 3.71 s, Bonferroni posthoc: ** *p* < 0.005). Unlike the males, the subchronic muscarinic antagonism in the females (18.68 ± 2.43 s, Bonferroni posthoc: ** *p* < 0.005) did not change with the TBI-induced reduction in time spent in the target quadrant. Subchronic donepezil reversed the TBI effect by increasing the time spent in the target quadrant by the female mice (29.77 ± 2.08 s, ^#^ *p* < 0.05; Figure 4(B2)).

The one-way ANOVA analyses revealed significant effects of the TBI and muscarinic modulation on the number of entries into the target quadrant in the male mice (F (3, 25) = 6.76; *p* = 0.001; Figure 4(A3)) but not in the female mice (F (3, 24) = 2.26; *p* = 0.106; Figure 4(B3)). Traumatic brain injury significantly impaired the spatial recall of the mice by reducing the number of entries into the target quadrant in the males (3.71 ± 0.56) when compared to the Sham mice (8.85 ± 0.88, ** *p* < 0.005). Subchronic scopolamine (8.25 ± 0.90) and subchronic donepezil (7.42 ± 1.06) significantly reversed the TBI-induced reduction in target quadrant entries in the male mice (^##^ *p* < 0.005 and ^#^ *p* < 0.05 respectively) (Figure 4(A3)). However, TBI alone or along with scopolamine or donepezil did not affect the number of entries into the target quadrant in the female mice when compared to the Sham group (Sham: 8.00 ± 0.37; TBI: 6.71 ± 0.80; TBI + Scopolamine: 6.42 ± 0.57; and TBI + Donepezil: 8.57 ± 0.53; Figure 4(B3)).

The one-way ANOVA revealed the significant involvement of muscarinic receptor activity and TBI in retrieving the position of the hidden platform in both the male (F (3, 25) = 5.344, *p* = 0.0055) and female (F (3, 24) = 3.986, *p* = 0.0195) mice (Figure 4(A4,B4)). TBI significantly impaired the recall of the platform location in the male (0.57 ± 0.36) and female (1.28 ± 0.52) TBI groups when compared to their respective Sham groups (male Sham: 5.14 ± 1.07, ** *p* < 0.005, Figure 4(A4); female Sham: 4.57 ± 1.02, * *p* < 0.05, Figure 4(B4)). Subchronic scopolamine (3.50 ± 0.94) and subchronic donepezil (3.42 ± 0.57) showed a slight enhancement in retrieving the platform location in the male mice (Figure 4(A4)). In contrast, in the females, subchronic scopolamine (2.14 ± 0.45) and subchronic donepezil (2.57 ± 0.64) had no effect on reversing the TBI-induced reduced platform location crossings (Figure 4(B4)).

### 3.4. Effect of TBI and Subchronic Cholinergic Modulation on Brain Edema

**Figure 5:**
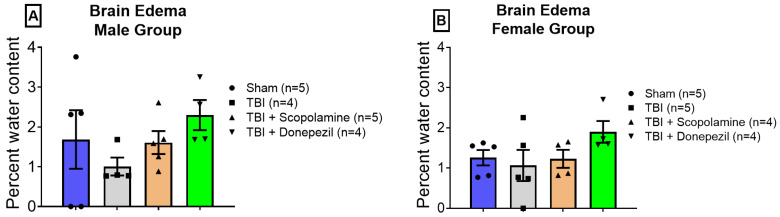
Effect of TBI and subchronic muscarinic modulation on brain edema. Graphs (**A**,**B**) represent the percentage water content in the brains of the male and female mice, respectively. Data are presented as mean ± SEM.

Brain edema or cerebral edema is the abnormal fluid accumulation and swelling of the brain in response to tumors [33], infection [34], toxin exposure [35], a lack of oxygen at higher altitudes [36], and trauma [37]. Edema induces hypoxic conditions in the brain by reducing the blood flow through the brain. We determined the effect of subchronic scopolamine and donepezil on TBI-induced brain edema. We did not find any significant difference in the whole brain water content in both the male (F (3, 14) = 1.059, *p* = 0.3976, Figure 5A) and female groups (F (3, 14) = 1.544, *p* = 0.246, Figure 5B).

## 4. Discussion

The neurological outcomes of mild TBI depend on the sex and cholinergic neurotransmission following a concussion [26,38]. In this study, we assessed the effect of muscarinic receptors on episodic memory retrieval after mild TBI in age-matched male and female mice. Our results revealed that acute muscarinic activation following trauma intensified the TBI-induced neurological dysfunction in males but not in females. Subchronic donepezil-induced neurological dysfunction in the male and female TBI mice. TBI-induced impaired spatial memory retrieval in all mice, irrespective of sex. Subchronic scopolamine and donepezil showed the potential to reverse TBI-induced spatial recall impairment in males. In contrast, fear memory retrieval was not affected by TBI or treatment in both sexes.

Previous studies have shown the time-dependent effects of scopolamine on TBI brains, showing neuroprotection immediately after injury and neurodegenerative effects when administered at a later time point [26,39,40]. This effect was witnessed in our experiment in a sex-biased manner, i.e., this response was only observed in the males (Figure 2(A1)). The female TBI mice displayed better recovery of neurological functioning following the trauma than the male TBI group, which supports previous reports showing the better resistance of females to TBI-induced neuronal damage than their male counterparts [41]. Though the underlying mechanisms of this sex-dimorphic neuroprotection to post-TBI outcomes in females are not completely understood, female gonadal hormones are reported to be associated with better neurological recovery following brain insult [41]. Following a TBI, females have exhibited decreased inflammation and enhanced neuronal survival [42,43,44].

A recent advancement in revealing the underlying cause of sex-dimorphic responses to post-TBI neuronal outcomes is reported by Doran et al. They showed the significant infiltration of proinflammatory cells in the brains of male mice compared to females. This differential neuroinflammatory process was suggested as a possible reason for sex differences in post-TBI neuronal outcomes and recovery [45].

In previous studies from our lab, we have reported that spatial memory retrieval was not affected by subchronic cholinergic modulation in females under physiological and chronic stress conditions [29,30]. A similar response was observed in the present study, where muscarinic modulation failed to show any effect in reversing TBI-induced spatial memory recall deficits in female mice. This suggests that spatial memory recall at relatively remote time points is independent of the muscarinic receptors in females under different retrieval conditions; however, further work is needed to confirm this hypothesis.

We did not find any effect of TBI in contextual fear retrieval in both sexes, which is inconsistent with the findings of Teutsch et al., who reported no effect of TBI alone in fear memory retrieval in male mice compared to controls [46]. In previous studies from our lab, we have reported a differential influence of subchronic donepezil and scopolamine on fear retrieval in a sex-dependent fashion. Donepezil enhanced the fear recall in males but showed the opposite effect in females [30]. Whereas subchronic donepezil administration under stress enhanced fear memory recall in mice, irrespective of sex [29]. We have also reported that contextual fear recall was independent of subchronic muscarinic antagonism in both sexes under physiological and chronic stress circumstances. At present, we are unable to propose the specific mechanism involved with these differential effects in fear retrieval among male and female groups. Further experiments that measure the inflammatory markers and endocrine hormones are needed to explain the influence of the inflammatory and endocrine mechanisms.

Female gonadal hormones are proposed to impart neuroprotective effects in younger and premenopausal women. Blaya MO et al. have comprehensively reviewed the female gonadal-hormone-dependent and independent variables that affect neurodegeneration, pathophysiology, and the outcomes of TBI in females across different phases of the female life span, supporting the importance of female gonadal hormones, i.e., progesterone and estradiol, and their neuroprotective effects in premenopausal females [47]. Gardner et al. reported, in their longitudinal cohort study, that there exists a significant interaction among post-mild-TBI-induced dementia in elderly females greater than 65 years of age [48]. Despite the importance of varying levels of female gonadal hormones at different stages of biological age in TBI neurological and cognitive outcomes, the majority of preclinical research has focused on using male subjects only. Considering the importance of the sex-dependent divergent outcomes of TBI and the influence of female biological age and life stage, using sex and lifespan stages (childhood, adolescence, pregnancy, pre-, and postmenopause) as critical variables in future TBI research might comprehend an understanding of the disease and treatment of those at a higher risk of dire outcomes.

Hippocampus theta oscillations are crucial for the formation and retrieval of episodic memories under physiological conditions [49]. Alterations to post-TBI cognitive and memory processes through alterations in brain electrical activity were assessed by Adamovich-Zeitlin et al. They recorded the neural biomarkers from the deep brain and cortical regions in a cohort of epilepsy patients with a TBI history, with a control cohort of non-TBI epilepsy patients. The EEG recordings of both groups showed similar patterns of theta frequency oscillations in memory encoding [50]. Future experiments on electrophysiological recordings of memory biomarkers will help toward developing potential treatments for TBI-induced memory dysfunctions through deep brain stimulation.

To the best of our knowledge, this is the first study that has compared the effects of muscarinic receptor modulation on episodic memory retrieval I n male and female mice under mild TBI. A limitation of current study is that the effect of muscarinic drugs on memory retrieval was assessed at a relatively early time point, following brain injury. Most of the cognitive outcomes of mild TBI occurs at later time points in the life of the subjects. Moreover, histological and synaptic changes should be studied in the future to draw better mechanistic insights for therapeutic purposes. Measuring hormone levels and additional studies into different severities of TBI should be carried out in the future. Further experiments are needed to study the effects of muscarinic receptor activity at the neuronal level to understand the mechanism behind this sex-dimorphic response in reversing mild-TBI-induced retrograde amnesia.

## 5. Conclusions

From our results we found that muscarinic receptors activity is essential to reverse TBI induced spatial memory retrieval impairment in males only, while fear memory retrieval is not affected by muscarinic modulation in mice of both sexes undergone TBI. Thus, the sex dimorphic effect of muscarinic receptors in reversing TBI induced retrograde amnesia depends on the type of memory.

## Figures and Tables

**Table 1:** Neurological severity score parameters and scoring paradigm.

S.	Task	Description	Maximum Score
1	Seeking behavior	Exploration of new environment by sniffing for 3 min	5
2	Beam balance	Motor coordination with balancing on a 7 cm-wide beam with steady posture for 10 s	3
3	Beam walk	Walking over 3 cm-, 2 cm-, and 1 cm-wide beams (80 cm long) within 2 min	3
4	Round stick balance	Motor coordination with hanging onto a stick (8 mm in diameter) for 10 s	1
5	Exit circle	Exiting a 30 cm diameter circle within 2 min	1
6	Straight walk	Motor coordination during walking on a straight surface	2
8	Hemi-monoparesis	Voluntarily holding onto forceps with hind limbs when lifted by tail	1
9	Acoustic startle	Bouncing/wincing to loud clap 10 cm above mice head	1
10	Wire suspension	Suspending from a 3 mm wire for 30 s	6
11	Pinna reflex	Head shaking in response to touching auditory meatus	1
12	Corneal reflex	Eye blinking upon touching cornea with cotton swab	1
13	Twisting	Twisting towards the tail when suspended 10 cm above a surface for 5 s	2

## Data Availability

Data will be made available on request.
